# Supplementary material for: Are carbon emissions trading and green financial instruments synergistic? -Comprehensive quantitative research based on content analysis
Source: PLoS One. 2024 Mar 7;19(3):e0298601. doi: 10.1371/journal.pone.0298601 (PMC10919601; doi:10.1371/journal.pone.0298601)
Supplement: S1 Data — (ZIP) [file pone.0298601.s001.zip › Data/地级市Z_F工作报告.zip!/║■─╧/╓Ω╓▐╩╨/└┤╫╘╣1⁄2╓┌║┼í╛┬φ┐╦╩2╛▌═°í┐_2011.docx]

各位代表：

　　现在，我代表市人民政府向大会作工作报告，请予审议，并请市政协各位委员和其他列席同志提出意见。

　　奋力跨越 超额完成“十一五”目标任务

　　过去五年，是株洲经济社会发展史上很不寻常、很不平凡的五年。五年来，我们在市委的正确领导下，以科学发展观统揽全局，围绕“保二争一、科学跨越”战略目标，成功抵御自然灾害、金融危机的强烈冲击，沉着应对建设高峰、偿债高峰的严峻挑战，充分把握“两型”社会建设综合配套改革试验区、中部崛起战略等历史性发展机遇，克难奋进，完成或超额完成“十一五”各项目标任务，全市经济社会发展跨越历史性的新台阶。

　　这五年，是经济结构不断优化、综合实力大幅提升的五年。全市GDP由2005年的521.6亿元增加到1274.8亿元，增长1.4倍，年均增长14.2%；财政收入由41.1亿元增加到130.9亿元，增长2.2倍，年均增长26%，是改革开放以来增长最快的时期；五年累计完成固定资产投资2252亿元，年均增长38.1%。非公经济占GDP比重由45%提高到60%，高新技术产业增加值从57.6亿元增加到259亿元，占GDP的比重达到20.3%，三次产业结构由2005年的13:50.5:36.5调整为9.7:58.5:31.8。轨道交通装备制造、有色冶金及深加工、汽车、陶瓷、服饰等产业集群不断壮大，涌现出3家过100亿元、4家过50亿元的龙头企业，全市工业增加值由225亿元增加到665亿元，增长1.95倍，新型工业化考核连续三年位居全省第二。园区产业集聚能力不断增强，园区规模工业增加值占全市规模工业增加值的比重达到53.7%。粮食年产量保持在185万吨以上，年出栏生猪达到440余万头，农产品加工企业发展到2700余家，加工产值超过150亿元。醴陵、攸县连续四年稳居全省县域经济十强县。社会消费品零售总额从181亿元增加到426.8亿元，年均增长18.7%。芦淞市场群进入全国十大服饰市场和服饰物流中心。中国驰名商标达到15件，被确定为国家商标战略实施示范城市。金融机构改革不断深化，金融体系日趋完善，新入驻股份制银行8家，获评中国金融生态城市。此外，房地产、现代物流、会展、保险、证券等服务业都有长足发展。

　　这五年，是基础设施继续完善、城乡面貌焕然一新的五年。大气魄推进重大基础设施建设。武广高铁客运专线、株洲西高铁站、醴潭高速、衡炎高速和长株高速竣工通车，实现“县县通高速”；杭长客运专线、长株潭城际铁路、衡茶吉铁路、浏醴高速、醴茶高速、茶界高速、分炎高速、炎睦高速、炎汝高速建设顺利推进；攸县煤电一体化项目开工建设，长株潭三市通信实现同号升位并网，天然气成功引入市区和醴陵。大手笔实施城市提质扩容。着力构筑“一体三极”格局，依托市区主体，向北打造云龙示范区，向西拓展河西新城，向南建设航空城；加快构建“一圈三环”路网，天元大桥、芦淞大桥建成通车，畅通了城市内环；中环大道东环北路开工建设，炎帝大道、时代大道、田心立交、王家坪立交等相继完工，荷塘大道开工建设，城市东西南北四张大门全部打开；体育中心、广电中心、规划展览馆投入使用，中心医院主体工程基本完工；强力实施“五改”工程，全力推进“四创四化”，城市功能日趋完善，城市品位明显提升，成功创建为中国优秀旅游城市、国家园林城市、国家卫生城市、全国交通管理模范城市。大力度完善农村基础设施。新建和改造干线公路205公里，建设乡村公路7645公里，基本实现“村村通水泥路”；投入资金124亿元，兴修水利工程15万处，治理病险水库275座，洮水水库基本建成；实施饮水安全工程，37万农民彻底告别了“喝水难”；在全省率先由政府支付农田灌溉水费；新建沼气池4.1万个；新造林76.3万亩，森林覆盖率达到61.5%；小城镇建设完成投资61.8亿元，城镇化率达到52%。炎陵县创建成国家卫生县城，并入选“中国十佳绿色城市”。

　　这五年，是“两型”建设率先突破、发展活力显著增强的五年。示范区建设全面铺开。云龙示范区完成投资45.6亿元，云峰湖国际旅游度假区、职教大学城、欧洲小镇等一批重大项目扎实推进；天易示范区拉开大河西发展框架，武广新城、栗雨中央商务区、湘水湾生态公园建设进展顺利，电动汽车、风电装备等战略性新兴产业项目相继开工；清水塘循环经济工业区着力“两改四提”，45个项目列入国家循环经济试点项目库，12个节能减排和5个限期治理项目稳步实施，产业正加速从“高碳”向“低碳”，从“制造”向“创造”转变。“两型”环境加速构建。大力开展“蓝天碧水净土静音”行动，关停污染企业123家，拆除工业烟囱284根，新建污水处理厂7座，实现城镇污水处理设施全覆盖；空气质量良好天数由298天提高到350天，湘江株洲段达到Ⅲ类水质标准，全市万元GDP能耗下降21.5%，规模工业增加值能耗下降61%。“两型”改革深入推进。按市场化运作理念，组建了城发集团、国投集团、地产集团等“6+2”投融资公司，公司法人治理结构逐步完善，经营意识逐步增强；城乡统筹改革和土地经营制度改革稳步推进，农村土地流转率达到33%，居全省第一；医药卫生体制改革、公立医院改革初见成效；省直管县财政体制改革、集体林权制度改革基本完成。对外开放不断扩大。进出口总额从6.9亿美元增加到14.7亿美元，年均增长16.3%。来株投资的世界500强企业达到10家，央企达到15家。

　　这五年，是社会事业全面进步、群众生活持续改善的五年。全市城镇居民人均可支配收入由11230元增加到19643元，年均增长11.8%；农村居民人均纯收入由3957元增加到7658元，年均增长14.1%。新增就业25万人次，农村劳动力转移就业年均稳定在68万人次，开发公益性岗位7133个，城镇零就业家庭动态为零，城镇登记失业率控制在4%以内；城镇居民医疗保险参保人数达71万人；新型农村养老保险开始试点；城区城镇居民最低生活保障线标准由每月170元提高到300元，始终保持全省领先水平；住房公积金发放贷款36.6亿元，支持城镇居民购房、建房440多万平方米；新增廉租住房1.5万套，新建经济适用房4545套，新建农村安居房5927栋。成功研制出我国首列高端A型地铁车辆；投入运营电动公交车达420台，是全国推广数量最多、运营里程最长的城市；第七次获评国家科技进步先进城市。大力推广“快乐德育、人文智育、阳光体育”，免费义务教育覆盖城乡，创建合格学校326所，新建城区中小学塑胶运动场52个，救助家庭贫困学生25万人次。甲型H1N1流感、手足口病等重大传染疾病防控取得明显成效。新型农村合作医疗参合率达到95.4%，社区卫生服务网络实现全覆盖；圆满承办省第十届体育运动会和2009年省艺术节。应急管理工作全面加强，防灾减灾能力不断提升。获评全省人口和计划生育模范城市，荣获“全国人民防空先进城市”称号，连续两年公众安全感和满意度民意测评居全省第一，连续两次荣获“长安杯”，连续四次荣获“全国双拥模范城”称号，连续五次被授予“全国民族团结进步模范集体”。与此同时，信访、妇女儿童、计划生育、广电、气象、市志、档案、国防、红十字、助残、老龄等事业都有新进步。

　　这五年，是政府职能加快转变、行政效能有效提升的五年。认真执行人大及其常委会决议、决定，自觉接受人大、政协和社会监督，共办结人大代表建议1317件，政协委员提案1436件，办结率、见面率均为100%，满意率99%以上。率先开展“法治株洲”创建，“大普法”、“大治理”格局基本形成。大力推进项目审批代理制、限时办结制、项目代建制等行政管理体制改革，行政审批事项减少69项。“数字株洲”建设步伐加快，政府信息公开深入推进,“12345”市长热线、市长信箱全面开通，妥善处理市民建议、投诉等事项5.2万件。行政监察、审计监督、廉政建设也取得新成效。

　　2010年是“十一五”的收官之年，我们按照“目标提高、标准提升、发展提速”的总体要求，紧扣转方式、促“两型”这条主线，着力打好“三大战役”，突出了四个重点。

　　一是突出打好“三大战役”。城市提质战果显著。四大精品工程完成投资35亿元，湘江风光带河西城区段、神农城广场对外开放，华强文化科技产业基地一期工程进展顺利，职教大学城已有7所院校入园。“四创四化”纵深推进，新增绿地面积481公顷，提质改造道路路面及人行道66条，亮化小街小巷33条，美化建筑物88栋，改造地下管网96处、老旧小区8个、门店橱窗200余栋，被列为国家数字化城管试点城市。创建国家环保模范城市26项指标已有21项达标。创建全国交通管理模范城市一举成功，获评“中国十大最具投资价值城市”。园区攻坚来势强劲。全年启动园区项目259个，固定资产投资达238亿元。南车株机公司实现销售收入159亿元，南车株洲所公司、株冶销售收入过100亿元。北汽集团南方基地年产20万辆整车项目成功下线，投资40亿元的变流技术产业园、投资50亿元的中航湖南通用航空发动机项目已经签约，山河智能通用航空产业项目正式落户。天桥起重成功上市，唐人神集团上市获批。旅游升温成效明显。旅游总人数达到1220万人次，实现总收入78亿元，增长30%；包装策划35个重点旅游项目，总投资578.7亿元，神农城、湘江风光带、荷塘月色、神农福地、酒埠江4A景区创建等项目全面启动，鹿原镇、酒埠江镇获评省特色旅游名镇。

　　二是突出推进“两型”建设。云龙、天易示范区和清水塘循环经济工业园规划编制全面完成。云龙示范区开发重大项目100余个，合同引资近1000亿元。云峰大道建成通车，迎宾大道基本建成，云龙大道全面开工。天易示范区高塘村完成土地改革试点，实现了“村组变社区、村民变居民、集体变国有”的历史性转变。完成霞湾港水变清一期工程、株冶总废水零排放工程，启动建设重金属污水处理厂和城市生活垃圾焚烧发电厂，“两型”环境建设初见成效。

　　三是突出统筹城乡发展。大力培育农业龙头企业，年产值过亿元的农产品加工企业达20家；实施“万村千乡市场工程”，新扩建农产品专业批发市场8家，新改建农贸市场21个、农家店600个；建设农业标准化示范基地48个。基本完成网株公路等4条干线公路改造，建成农村公路1135公里；建设生活垃圾处理系统356个，20万农户用上清洁能源；整理土地80多万亩，农业机械化率达60%。新增农村劳动力转移就业6.9万人；1.8万名农村“五保”老人得到供养，10.5万农民领到低保救济金。

　　四是突出改善民生民利。新增城镇就业5.1万人，失业人员再就业2.6万人。建成义务教育合格学校20所，市区新建学校3所，新增学位4860个，资助家庭贫困学生3.57万人。在城市四区及株洲县实现药品零差价销售，就诊药品费用下降38%。扩大新型农村养老保险试点，新增参保人数26.1万人，企业养老保险新增参保人数8.8万人。新增廉租住房7115套，新建公共租赁房470套，新建农村安居房1100栋。全市1.3万户、67万平方米的棚户区改造全面启动。十件民生实事全面完成（见附表）。

　　各位代表，过去五年，我们始终坚持“保二争一、科学跨越”的战略目标不动摇，致力推进新型工业化、新型城镇化、农业现代化、信息化，发展思路更加清晰；我们始终坚持“以现代工业文明为特征的生态宜居城市”的发展定位不动摇，加快转变发展方式，着力调整产业结构，发展质量全面提升；我们始终坚持“内提外拓、统筹发展”的城市发展格局不动摇，“一体三极、一圈三环”城市框架初步构建，城市空间全方位拓展，发展后劲明显增强；我们始终坚持“以人为本、关注民生”的执政理念不动摇，坚定不移地实施惠民行动，想群众所忧，急群众所盼，解群众所难，切实做到发展依靠人民、发展造福人民，发展成果全民共享。五年风雨，五载耕耘，我们真切地体会到，做好政府工作，必须坚持解放思想，推进改革创新；必须坚持科学发展，注重统筹兼顾；必须坚持以人为本，重视民生民利；必须坚持激情工作，做到规范管理；必须坚持勤政廉政，严格依法行政。

　　发展的成绩来之不易，积累的经验弥足珍贵，创造的精神影响深远。这是市委正确领导、市人大和市政协监督、支持的结果，是各民主党派、工商联、无党派人士、各人民团体参政议政的结果，饱含了各位代表和委员的智慧力量，凝聚了全市人民的辛勤汗水。在此，我代表市人民政府，向各位人大代表和政协委员，向驻株部队官兵和全市人民，向关心、支持、参与株洲建设的各界人士，致以崇高的敬意和诚挚的谢意！

　　在总结成绩的同时，我们也清醒地看到，我市经济社会发展中还存在不少困难和问题，主要是：解放思想的力度还不大，实现“两型”改革率先突破的办法不多；经济总量不够大，产业结构不够优，转方式、调结构任务依然繁重；制约经济增长的资金、土地、人才等要素瓶颈有待破解，环境保护的压力仍然较大；项目策划和包装水平不高，推动跨越发展的大项目、好项目不多，发展后劲有待增强；统筹城乡发展任务艰巨，农民增收渠道有待拓宽；一些涉及群众切身利益的问题亟需解决，维护社会稳定的压力较大；政府自身建设需要继续加强，执行力、创新力有待进一步提升；等等。对此，我们将高度重视，认真研究，采取有力措施，切实加以解决。

　　奋勇前行 科学谋划“十二五”宏伟蓝图

　　“十二五”时期，是株洲大有作为的机遇期，是基本实现全面小康的关键期，是“两型”社会建设的攻坚期，是推进新型城镇化的加速期。未来五年，国内经济发展方式将发生重大转变，发展难度不断加大，但国家重大战略布局的实施和全省“四化两型”战略的推进，为株洲发展创造了难得的历史机遇。我们既要抓住机遇、用好机遇，又要应对挑战、战胜挑战，以强烈的事业心和使命感，争创新优势，推进新跨越。

　　“十二五”时期政府工作的指导思想是：高举中国特色社会主义伟大旗帜，坚持以邓小平理论、“三个代表”重要思想为指导，深入贯彻落实科学发展观，以加快转变发展方式为主线，围绕“以现代工业文明为特征的生态宜居城市”的定位，全面推进“四化两型”建设，深入持久打好城市提质、园区攻坚和旅游升温“三大战役”，大力实施科教先导、产业转型、城镇带动、民生优先战略，努力建设智慧株洲、实力株洲、绿色株洲、幸福株洲，实现经济社会又好又快发展，基本建成全面小康社会和“两型”社会示范区。

　　全市经济社会发展的总体目标是：“两个基本建成、三个翻番、两个明显下降”，即到2015年，基本建成全面小康社会、基本建成“两型”社会建设综合配套改革示范区，全市GDP、财政收入和城乡居民收入翻一番，万元GDP能耗下降15%、主要污染物排放量下降10%。GDP年均增长13%以上，达到2500亿元以上，三次产业结构调整到5:55:40；财政总收入年均增长15%以上，达到260亿元；居民消费率达到40%；固定资产投资年均增长20%；进出口总额年均增长13%；城乡居民收入年均增长14%；人口自然增长率控制在8‰以内。努力建成全国环境保护模范城市、国家生态园林城市、国家创业型城市、全国文明城市。

　　围绕上述指导思想和发展目标，我们将紧扣科学发展这一主题，突出转变发展方式这条主线，按照“两型”理念、国际视野、战略思维和务本求实的要求，转方式、促“两型”、保增长、惠民生，纵深推进“两型”社会建设，深入持久打好“三大战役”，大力实施“四大战略”，努力建设“四个株洲”，全面推动经济社会新跨越。

　　（一）实施科教先导战略，建设智慧株洲

　　加快构建完善的科技创新体系、现代教育体系、信息应用体系，形成强劲的创新发展能力、教育支撑能力、智慧管理能力，打造智慧株洲。

　　实施科技创新工程。充分发挥信息技术对科技创新的引领作用，抓好科技平台建设，强化企业创新主体地位，努力打造国家创新型城市。大力推进自主创新，力争在轨道交通、电动汽车等领域实现科技攻关的重大突破。加速科技成果转化，发展高新技术企业200家以上，争取实施50项以上国家“863”计划，新建10个以上国家级和省级工程实验室，力争高新技术对经济增长的贡献率提高到60%，高新技术产业增加值占GDP的比重提高到30%以上。

　　实施教育提升工程。抓紧实施《株洲市建设教育强市行动计划》和《株洲市城区基础教育三年攻坚计划》。均衡发展义务教育，优化资源配置，改善办学条件。优先发展职业教育，加快职教大学城建设步伐，积极构建职业教育“立交桥”，努力把株洲打造成为“中部地区职业教育创新之都”。重点发展高等教育，支持湖南工业大学做大做强。统筹发展学前教育、高中教育、成人教育、特殊教育，构建较为完备的国民教育体系，建设教育强市。

　　实施信息应用工程。加强信息基础设施建设，实现高速宽带城域网覆盖全市，推进“三网融合”，抓好电子商务、物联网和云计算的建设与推广，构建信息高速网络平台。加快建设新一代“数字株洲”电子政府，打造全市统一的政务网络平台和公众信息服务平台。继续完善数字城管、治安电子防控和智能交通管理系统，抓紧建设城市规划、医疗、社保、环保、统计、计生、民政等信息管理子系统，构建高效、现代化的公共管理指挥平台。不断完善智慧服务系统，建立集住房、教育、水电气供应、应急处置、社会治安等功能于一体的智慧应用体系。

　　（二）实施产业转型战略，建设实力株洲

　　做强传统产业，做优新兴产业，做大经济总量，优化产业结构，着力形成优势产业高端化、传统产业优质化、新兴产业规模化，构建具有核心竞争优势的“两型”产业体系，打造实力株洲。

　　加快推进新型工业化。继续实施“5115”工程，支持南车株机公司、南车株洲所公司、南车电机公司、株冶集团、株硬集团、北汽集团、中航南方、唐人神等企业继续做大做强，真正成为具有强劲引领和带动力的旗舰企业。深入实施中小企业成长工程，着力搭建融资、配套协作、技术创新和信息服务平台，解决中小企业发展难题。持续打好园区攻坚战役，进一步推进园区基础设施建设，破解土地、资金等要素瓶颈，优化园区管理和服务，加速产业向园区集中，园区向特色发展。突出轨道交通千亿产业园区建设，着力培育汽车千亿产业集群，推进八大特色专业园区建设，构筑工业高速发展的坚实平台。建成1个总产值过100亿元、4个总产值过50亿元的县域工业园区。认真落实“6+1+4”产业发展行动计划，改造提升交通装备制造、有色冶炼、化工、食品加工、陶瓷、烟花等传统优势产业，大力发展新能源、新材料、航空航天、生物医药等战略性新兴产业，加快建成“四城三基地”，即轨道科技城、航空城、汽车城、服饰城和有色金属新材料精深加工基地、新能源产业基地、中药现代化和健康食品产业基地，再造一个株洲工业。

　　努力实现农业现代化。坚持“两跳”方针，跳出“农”字抓农业，以工业化理念发展现代农业，引导农产品加工企业进园区，培育一批年产值过100亿元、50亿元的农产品加工企业，五年内产值翻一番；跳出农业抓农村，以城镇化的理念建设新农村，切实提高城镇规划水平，加大城镇投入，做大城镇规模，提高城镇品位。强化“两调”举措，以调整农业产业结构为重点，不断调低农业在三次产业中的比重；大力发展生态、休闲、体验、观光等现代农业，努力调高现代农业在整个农业中的比重。加快“两转”步伐，加速推进农村土地流转，引导农业产业向规模发展集中、农民向城镇和新型社区集中、土地资源向土地资本集中；实施“百万农民进城工程”，大力推进农业产业化，积极发展劳务经济，引导农民向城镇转移，向产业工人转变。

　　大力提升现代服务业。继续打好旅游升温战役，加强策划宣传，深化区域合作，打造精品线路，加快“一廊两片”、“一城三湖”、“一江八镇”建设步伐，推进炎帝陵创建国家5A景区、湘江风光带、云峰湖国际度假区、云阳山、酒埠江、荷塘月色等重大旅游项目的开发建设，精心打好“炎帝牌、红色牌、生态牌、工业牌”等特色旅游品牌，努力把株洲建成全国知名旅游目的地，力争旅游业产值占GDP比重达到7%以上。加快现代物流业发展，组建一批大型专业物流企业，建成长株潭国际物流园、芦淞现代服饰物流仓储配送中心、荷塘生活用品商贸城，努力构建面向全省、辐射中南地区的区域性物流中心。加强房地产市场引导调控，促进房地产业健康发展。加大招商引资力度，一手抓存量扩张，一手抓增量引进，在做大做强现有企业的同时，大力引进战略投资者。推进芦淞服饰市场群提质改造，扶持大型商业超市连锁经营，完善商贸流通体系，提升消费水平。推进金融生态环境建设，促进政银企交流对接，充分发挥金融对经济的支撑作用。积极发展信息咨询、会展、电子商务、服务外包、文化创意等新型服务业，打造一批服务业品牌。

　　（三）实施城镇带动战略，建设绿色株洲

　　科学制定城镇规划，调整优化城镇布局，加快城镇提质扩容步伐，增强城镇集聚和辐射能力，致力形成城镇相连、交通便捷、生态宜居的“两型”城镇体系，打造绿色株洲。

　　加快构建新型城镇体系。中心城市按照“两型”理念和“一体三极、三环七射”的战略构想，强力推进云龙示范区、天易示范区、航空城建设，进一步拉开城市发展框架。加快建设武广新城、金山新城、新马片区、白井片区，实施“一江八镇”城市拓展建设工程，力争2015年建成区面积达到150平方公里。深入持久打好城市提质战役，纵深推进城市创建工作，健全城市管理长效机制。抓好城区污水处理、停车场所、公交站场、消防设施、垃圾处理等市政设施建设，完善电力、自来水、燃气、新能源等供应保障设施，强化城市功能。按照“一主两次、两轴四带”的发展构想，以株洲市区为主中心，以醴陵市区、攸县县城为次中心，沿106国道、S211省道完善市域城镇体系，加快推进小城镇建设，着力提升县城在县域经济中的首位度。

　　加快构建交通能源体系。构筑大交通，建设大枢纽，形成铁路、公路、水运、航空等多种交通运输方式相互衔接、高效便捷的综合交通运输体系。建成沪昆高速客运专线、衡茶吉铁路株洲段，加快长株潭城际铁路株洲段建设，改造提升醴茶铁路。建成岳汝、泉南、分炎、炎睦、长株潭南环等高速公路，完成京港澳高速株洲段和左权大道等拓改工程，力争建成长株潭高速东环线，形成市域2小时高速通勤圈。建成洞株路、铜霞路等城际干道；开工建设湘江大道、长株攸快速路株洲段，完成芷渌公路、G106、S320等7条干线公路改造。完成湘江株洲段航道改造工程，建成铜塘湾新港区，使湘江主航道常年达到2000吨级航道标准。建成株洲通用机场，形成比较完善的区域立体交通网络。加快攸县煤电一体化项目、茶陵洮水电站、攸县桐坝电站等新增电源点的建设。完成“西气东输”二线工程，构建稳定、清洁、安全的能源供应体系。

　　加快构建绿色生态体系。按照“人在林中、城在园中、路在景中”的目标，扎实推进湘江防护林、退耕还林、生态公益林、“三边”造林等项目建设，确保森林覆盖率稳定在62%以上。以创建国家生态园林城市为抓手，完善城市绿地系统规划，抓好城区生态园林建设，增加城市绿化量，构建立体化的城市绿化系统，城区绿化覆盖率达到50%以上。

　　加快构建环境保护体系。继续开展“蓝天碧水净土静音”行动，加快淘汰落后产能，重点治理工业污染源。加强“一江四港”综合治理。推进清水塘地区环境污染综合整治，加快重金属污染土壤的生态修复和综合利用，加快建成重金属污水处理厂，彻底改变清水塘地区面貌，创建国家环保模范城市。落实国家耕地保护制度，确保跨区域占补平衡；严厉打击非法开采、乱采滥挖矿产资源行为，深入推进矿区环境综合治理。加强重点行业和企业节能工作，加大可再生能源建筑应用推广力度，倡导绿色消费，打造低碳示范城。扎实推进城镇、农村治污工作，构建生态宜居家园。

　　（四）实施民生优先战略，建设幸福株洲

　　高度关注民生，切实改善民生，努力解决人民群众最关心、最直接、最现实的利益问题，让人民群众共享发展成果，构建幸福株洲。

　　实施收入倍增工程。把促进就业作为保障和增加城乡居民收入的根本举措，努力拓宽就业渠道，通过大力发展经济带动就业，实现城乡比较充分就业。完善收入分配统筹机制，稳步提高机关事业单位和离退休人员工资水平。积极引导企业建立工资增长机制，不断提高企业职工劳动报酬。加强市场价格监控，努力保持物价基本稳定。加强农民就业培训，拓宽农民增收渠道，落实各项惠农政策，提高农民收入水平。

　　实施社保全覆盖工程。全面推行新型农村养老保险制度，建立健全城乡一体化的基本医疗保障制度，完善农民工社会保险制度，打造“广覆盖、多层次、可持续”的社会保障体系。加强社会救助体系建设，适度提高最低生活保障水平，增强社会救助能力，努力实现城乡居民人人享有社会保障。构建廉租住房、公共租赁住房和农村安居房等多层次住房保障体系，完成棚户区改造和“城中村”整治任务，推进非公组织公积金制度建设，努力改善中低收入居民的住房条件。

　　实施健康惠民工程。加强医疗卫生机构基础设施建设，落实市、县、乡、村（社区）四级医疗卫生机构建设项目，建成市中心医院并投入使用，构建结构合理、布局均衡、覆盖城乡的医疗卫生服务体系。落实国家基本药物制度，实施食品放心工程，建立健全食品药品供应和安全保障体系。推进公立医院改革，整合城区医疗资源，组建各具特色的医疗联合体，提高医疗服务质量。继续完善公共卫生体系，强化公共卫生和重大疾病防控工作，不断提高群众健康水平。

　　实施文化提升工程。突出抓好图书馆、博物馆、科技馆、大剧院、艺术中心等城市公共文化设施建设，让市民享受更高品质的现代城市生活。加快推进乡镇综合文化站和农家书屋等公共文化设施建设，构建比较完善的公共文化服务网络。创作一批艺术精品，举办系列重大节日文化活动，活跃人民群众文化娱乐生活。加快建成华强文化产业基地，发展壮大文艺演出、新闻出版等文化产业，推动文化事业大发展大繁荣。加强公共体育设施建设，推进全民健身运动，积极承接高规格高水平的体育赛事，打造有影响力的城市体育品牌。

　　奋起赶超 努力实现“十二五”良好开局

　　2011年，是“十二五”的开局之年，也是株洲建市60周年。抓好今年的工作，对于全面完成“十二五”目标任务至关重要。今年全市经济社会发展的主要预期目标是：GDP增长13.5%；财政总收入和一般预算收入增长13.5%；固定资产投资增长30%；社会消费品零售总额增长18%；力争城乡居民收入增长14%；万元GDP能耗下降5%以上，主要污染物排放总量下降3%以上；城镇登记失业率控制在5%以内；居民消费价格指数在4%以内；人口自然增长率控制在7.5‰。

　　为实现以上目标，我们将按照“保增速、保提质、保民生”的总体要求，加快转变发展方式，继续打好“三大战役”，突出抓好十大产业项目、十大基础工程、十大民生实事，努力推动经济社会又好又快发展，为“十二五”开好头，起好步。

　　十大产业项目：1.加快轨道千亿产业园区建设力度，重点推动时代研究院、第二制造中心、动车组等项目建设，着力完善园区基础设施。2.开工建设五矿集团有色金属新材料精深加工基地，提升传统冶炼产业。3.启动通用航空机场、通用航空发动机和轻型飞机等项目建设，着力打造国家航空高技术产业基地。4.推进北汽集团南方基地二期续建工程，为打造千亿汽车产业集群奠定基础。5.加快推进风电装备产业化进程，着力打造新能源产业基地。6.加快推进唐人神集团1000万头生猪产业链一体化工程建设，提升农业产业化水平。7.积极推进旗滨玻璃二期工程建设，提升现代建材产业水平。8.推进攸县煤电一体化项目建设，力争完成电厂厂房一期工程。9.加快推进湖南华强文化科技产业基地建设，确保年内开园营业。10.推动南车时代与曙光汽车合作，建设新能源客车基地。

　　十大基础工程：1.加速神农城建设，打造全球华人炎帝文化景观中心。2.加快推进湘江风光带建设，基本建成湘江河西防洪景观工程。3.启动“一江四港”整治工程，建成重金属污水处理厂，有效解决清水塘污染问题。4.完成铜塘湾港区主体工程，提升湘江港口吞吐能力。5.启动湘江六桥建设，完善主城区交通体系。6.启动湘江大道建设前期工程，逐步形成大河西发展格局。7.开工建设航空大道，基本完成荷塘大道主体工程，加快“一体三极”格局的形成。8.加快浏醴、醴茶、茶界、分炎、炎汝、炎睦等高速公路建设，构建市域2小时高速通勤圈。9.加快职教大学城建设步伐，开工建设7所院校，5所竣工投入使用。10.完成神农大剧院和神农艺术中心主体工程，启动图书馆和科技馆的建设，提升城市品位，丰富市民生活。

　　十件民生实事：1.积极扩大就业。新增城镇就业4.8万人、失业人员再就业2.2万人、“零就业家庭”动态援助100％、新增农村劳动力转移就业6万人。2.基本完成公立医院改革试点工作，切实解决看病难、看病贵的问题。新型农村合作医疗参合率巩固在90%以上，新农合政策范围内住院费用平均补偿率达到60%；城镇居民医疗保险住院补偿率稳定在53%以上，新建或改造社区卫生服务中心20所，建成中心医院并投入使用。3.改善就学条件。建成义务教育合格学校68所；资助家庭贫困学生1.85万人；完成城区基础教育扩容提质工程项目27个，启动市二中、白鹤小学武广新城分校建设。4.加强住房保障。新增廉租和公租住房6500套；改造国有林场危旧房865户；改造农村危房1000户；新增廉租住房租赁补贴家庭800户；住房公积金覆盖率达到85%。5.完善社保体系。稳定新型农村养老保险参保人数50万人；新增企业养老保险参保人数3.66万人，稳定城镇居民医疗保险参保人数69万人。6.保护生态环境。加大“一江四港”治理力度，确保湘江株洲段保持国家Ⅲ类以上水质，加大清洁能源推广和大气污染综合整治，确保空气质量达到二级；城市污水处理率、垃圾无害化处理率分别达到85%、100%；农村建设沼气池6000口；新增混合动力公交车207台。7.完善农贸市场。城区新建改造农贸市场12个，每个县市至少完成1个示范点。8.基本建成公共自行车租赁系统。9.加强社会救助。城区城镇低保保障线标准提高到330元，农村提高到65元，农村五保户分散供养标准提高至1600元/年，新建和改扩建敬老院4所。为困难群众实施法律援助案件1200件。10.保障食品药品安全。每年投入1000万元，扶持建设一批高标准无公害蔬菜基地。健全食品药品质量安全监管体系，确保各类食品抽检平均合格率达到95%以上；蔬菜农药残留超标率控制在8%以内；餐饮业餐饮服务许可证持有率达到100%；基本药物目录考核品种质量合格率98%以上；新解决11.5万农村人口饮水不安全问题。

　　重点抓好以下五个方面的工作：

　　（一）加快转变方式，提升发展质量。继续打好园区攻坚战役，重点抓好“十百千”工程，建好栗雨工业园、董家塅高科园、田心高科园、金山工业园、南洲工业园、醴陵陶瓷产业园、攸州工业园、金星工业园、九龙工业园和云龙创意产业园等十个产业园区；培育航空装备产业园、风电产业园、变流及电子信息产业园、钨钼铅锌精深加工产业园、纺织服饰产业园、釉下五彩创意园、电瓷电器园等百亿特色产业园区；加快推进轨道交通千亿产业园区和汽车及零部件千亿产业集群建设步伐。大力发展现代农业，稳定粮食生产面积在380万亩以上，提高粮食综合生产能力；加速农村土地流转，推进农业标准化建设，争创2个省级和8个市级农业标准化示范区；稳步推进茶陵现代烟草农业建设，扩大农产品精深加工，力争培育2个省级和10个市级龙头企业。努力扩大内需，完成芦淞服饰城综合提质改造工程，推动芦淞商圈新一轮发展；落实国家财政补贴政策，继续推进“家电下乡”、“以旧换新”工作；启动株洲烟草物流中心、荷塘生活用品商贸城、中南粮油交易中心等项目建设。继续打好旅游升温战役，重点抓好“123”工程，即办好以湖南旅游节为龙头的系列旅游节会活动；建设岳汝高速株洲段沿线和“醴陵仙岳山-婆仙岭-仙庾岭-云峰湖”城郊两大旅游长廊；突出抓好神农福地、云峰湖国际度假区、神农城三个重大项目；弘扬炎帝文化，办好辛卯年省长公祭炎帝陵活动，全力打造“炎帝五千年，中部欢乐谷”精品旅游品牌。

　　（二）加快“两型”建设，凸显示范效应。加速示范区建设步伐，云龙示范区抓紧建成云龙大道、华强路，加快推进云峰湖体育公园、职教大学城、欧洲小镇等项目建设；加快天易示范区栗雨中央商务区、武广新城、新马片区等项目开发建设。加强环境污染治理，加大“一江四港”综合整治力度，尽快启动建设白石港生活污水处理厂，完成南郊垃圾处理场渗漏液处理设施改造工程、建宁港和霞湾港清淤及港体整治工程；加快生活垃圾焚烧发电厂和城镇生活垃圾处理场建设；积极开展全国节水型试点城市创建工作，着力推进节能减排全覆盖工程，严格落实节能减排考核目标责任，引导企业加大污染治理和节能减排投入，狠抓重点工业污染源治理，突出抓好清水塘地区污染治理，确保清水塘重金属污水处理厂如期完工，做好市区洗水企业搬迁改造工作。加快推进“两型”改革，按照“资源资产化、资产资本化、资本证券化”的思路，深化投融资体制改革，发挥各类投融资公司的主体作用，为城市建设和产业发展提供更有力的支持；加快土地经营制度改革进程，启动耕地异地占补平衡、农村宅基地退出补偿和流转试点工作；启动主要污染物排污权交易改革；启动水务一体化改革，整合涉水事务管理职能；继续深化城区建设体制等领域的改革，力争取得新的突破。

　　（三）加快招商引资，增强发展后劲。以开展“招商引资年”活动为抓手，健全工作机制，扩大招商成效，确保全年完成招商引资任务200亿元，力争突破300亿元。瞄准世界500强、国内200强等大集团、大财团和战略投资者，策划包装一批对全市经济社会有重大影响的大项目。立足传统优势产业和新兴产业，大力开展以商招商、上门招商、节会招商和特色园区招商，积极参加“粤洽周”、“沪洽周”等招商活动，引进一批符合国家产业政策的大项目、好项目。全力抓好全球轨道交通发展论坛、全球华商峰会暨炎帝服饰节等会展招商活动，进一步扩大招商影响力，增强招商实效。加大资本市场融资力度，积极引进PE投资（私募股权投资）和风险投资。同时，着力优化对外贸易结构，鼓励有条件的企业“走出去”，广泛开展对外交流合作，大力发展服务外包和加工贸易，致力打造以湖南华强和时代电气轨道交通为核心的服务外包示范园。

　　（四）加快城乡统筹，促进协调发展。继续打好城市提质战役，重点抓好“448”工程，即深入推进“四创四化”，加快建设神农城、湘江风光带、职教大学城、华强文化科技产业基地等“四大精品工程”；积极稳妥地构建有利于城市发展的八大体系；启动智慧城市建设，加快推进“三网融合”步伐；动工兴建湘江六桥，全面建设自行车专用绿道；加快老旧小区水电改造。完善城乡基础设施，推进长株潭城际铁路、衡茶吉铁路、杭长客运专线等重大基础设施建设。建成分炎、炎睦高速公路，加快建设岳汝高速株洲段、茶界高速，不断完善区域交通网。建成S315龙下至攸县段、S322炎陵至牛岗排段、S211株洲段、G106炎陵至槽里段和芷渌公路。启动炎帝陵至神农谷、云峰湖至官庄水库等旅游环线建设。加大农村基础设施投入，新建农村公路1000公里，抓好新农村示范工程和示范片建设，进一步改善农村生产生活环境。抓紧云田、松西子、仙庾等城乡统筹改革试点，进一步巩固试点成果；稳步推进户籍制度改革，实现城市四区和云龙示范区村民变居民；着力开展农村产权制度改革，推动农村集体建设用地使用权流转，健全集医疗、养老、低保于一体的城乡保障体系，积极推进统筹城乡就业试点，实现农村劳动力转移就业70万人；启动村级治理制度改革，扩大农村社区建设试点范围，力争覆盖率达到30%。

　　（五）加快民生改善，构建和谐社会。继续完善保障体系，优化创业环境，推进就业创业指导中心建设，努力使我市成为要素集聚、功能配套的区域性创业中心，打造国家创业型城市；巩固新型农村养老保险试点成果，逐步在全市推行；加大保障性住房建设，启动廉租房“荷花家园”小区建设，兴建一批公共租赁住房。大力发展社会事业，加快建设轨道交通国家工程技术中心，全面完成公交车电动化三年行动计划；切实抓好各级各类教育，创建6所优质初中，30所乡镇中心幼儿园；加强重大传染疾病预防控制，完成20个社区卫生服务中心规范化建设；加快建设一批无公害蔬菜基地，加大食品市场整治力度，确保食品安全；加强价格执法，确保物价指数控制在全省平均水平以下；办好建党90周年、建市60周年庆祝活动。切实维护社会稳定，完善突发事件应急管理机制，全面提高基层应急救援能力；做好“六五”普法工作，推进依法治市进程；强化劳动保障监察执法，维护劳动者合法权益；加强社会救助、社会福利、慈善和红十字工作；全面落实安全生产责任制，严防重特大事故发生，加强社会治安综合治理，严厉打击各种违法犯罪活动，巩固“平安株洲”创建成果；提升全民国防意识，加强国防动员和民兵预备役部队建设，争创“双拥模范城”五连冠。

　　奋发有为 全力保障“十二五”跨越发展

　　实现“十二五”目标任务，我们必须落实“五个敢于”的发展理念，以更前瞻的思维，更超人的胆略，更优质的服务，更务实的政风，为经济社会又好又快发展提供坚强保障。

　　着力推进体制创新。创新行政管理体制。按照“精简、统一、效能”的原则，进一步加快政府职能转变，整合政府机构，优化政务流程，加快形成权责一致、决策科学、执行顺畅的管理体制。创新经济发展体制。紧扣转变发展方式这一主线，围绕产业升级、自主创新、统筹城乡、节能减排等重点领域，加大政策扶持力度，破解劳动力、土地、资金、市场、价格等要素瓶颈，努力形成地尽其利、物尽其用、货畅其流的良好格局。创新社会管理体制。加快收入分配、社会保障、医疗教育等社会事业领域改革，增强发展活力，提升保障水平，使人民群众生活更有尊严、更加幸福。

　　着力优化发展环境。弘扬“火车头精神”，提升城市精神状态，追求卓越，勇创一流，营造开放包容的人文环境。按照建设“法治湖南”的要求，全面推进依法行政，促进决策的制度化、科学化、法制化，规范行政执法权力，构建规范健全的法制环境。大力整顿市场经济秩序，加大反不正当竞争行为力度，严肃查处各种仿冒、欺诈行为，切实维护广大企业和消费者的合法权益，打造公平有序的市场环境。按照公开、透明、精简、统一的原则，制定更加优惠的政策，简化行政审批事项，减少审批环节，优化审批流程，提高审批效率；深入治理乱收费、乱检查、乱罚款、乱摊派，切实减轻企业和群众负担，建立宽松开明的政策环境。

　　着力打造人才高地。加强人力资源开发，设立人才培养基金，着力培养一批先进制造业、现代农业、医药食品、现代物流等产业发展的紧缺人才；加强企业家队伍建设，培养一批高素质的现代企业管理人才。开辟“绿色通道”，加快高端人才引进，突出抓好博士后科研工作站、企业研发中心以及科技工业园等各类人才基地建设。加大对各类优秀人才的奖励力度，改革企事业单位内部收入分配制度，鼓励专利、发明、技术、资金、管理等生产要素参与收益分配，逐步建立重实绩、重贡献、向优秀人才倾斜的分配激励机制。放宽人才政策，提高人才服务水平，努力营造人才荟萃、人尽其才的良好氛围。

　　着力加强政府建设。自觉接受同级人大及其常委会的监督，贯彻执行人大及其常委会的决议、决定，积极支持人民政协发挥政治协商、民主监督、参政议政的作用，认真办理人大代表建议和政协委员提案。建立和完善重大决策调研制度、专家咨询论证制度和社会公示听证制度，提高政府决策的科学化、民主化水平。妥善处理经济发展与社会和谐的关系，排查化解人民内部矛盾，切实维护群众利益；改进市长热线和信访工作，畅通群众利益诉求渠道，认真解决群众反映强烈的热点难点问题。全面落实党风廉政建设责任制，严格执行国有资产监管、财政、审计、监察等制度，切实加强廉政建设，主动接受法律监督、社会监督和舆论监督，提高拒腐防变能力，努力打造一支清正廉洁、作风过硬的公务员队伍。

　　各位代表，站在新的起点，迈上新的征程，我们信心百倍，激情满怀。让我们在市委的坚强领导下，同心同德，开拓创新，扎实工作，奋发图强，为实现“十二五”宏伟目标，全面建设智慧、实力、绿色、幸福的新株洲而努力奋斗！
